# Toward Optimal Heparin Dosing by Comparing Multiple Machine Learning Methods: Retrospective Study

**DOI:** 10.2196/17648

**Published:** 2020-06-22

**Authors:** Longxiang Su, Chun Liu, Dongkai Li, Jie He, Fanglan Zheng, Huizhen Jiang, Hao Wang, Mengchun Gong, Na Hong, Weiguo Zhu, Yun Long

**Affiliations:** 1 Department of Critical Care Medicine Peking Union Medical College Hospital Peking Union Medical College & Chinese Academy of Medical Beijing China; 2 Digital China Health Technologies Co Ltd Beijing China; 3 Department of Information Management Peking Union Medical College Hospital Peking Union Medical College & Chinese Academy of Medical Beijing China; 4 Department of General Internal Medicine/Department of Information Management Peking Union Medical College Hospital Peking Union Medical College & Chinese Academy of Medical Beijing China

**Keywords:** heparin, dosing, machine learning, optimization, intensive care unit

## Abstract

**Background:**

Heparin is one of the most commonly used medications in intensive care units. In clinical practice, the use of a weight-based heparin dosing nomogram is standard practice for the treatment of thrombosis. Recently, machine learning techniques have dramatically improved the ability of computers to provide clinical decision support and have allowed for the possibility of computer generated, algorithm-based heparin dosing recommendations.

**Objective:**

The objective of this study was to predict the effects of heparin treatment using machine learning methods to optimize heparin dosing in intensive care units based on the predictions. Patient state predictions were based upon activated partial thromboplastin time in 3 different ranges: subtherapeutic, normal therapeutic, and supratherapeutic, respectively.

**Methods:**

Retrospective data from 2 intensive care unit research databases (Multiparameter Intelligent Monitoring in Intensive Care III, MIMIC-III; e–Intensive Care Unit Collaborative Research Database, eICU) were used for the analysis. Candidate machine learning models (random forest, support vector machine, adaptive boosting, extreme gradient boosting, and shallow neural network) were compared in 3 patient groups to evaluate the classification performance for predicting the subtherapeutic, normal therapeutic, and supratherapeutic patient states. The model results were evaluated using precision, recall, F1 score, and accuracy.

**Results:**

Data from the MIMIC-III database (n=2789 patients) and from the eICU database (n=575 patients) were used. In 3-class classification, the shallow neural network algorithm performed the best (F1 scores of 87.26%, 85.98%, and 87.55% for data set 1, 2, and 3, respectively). The shallow neural network algorithm achieved the highest F1 scores within the patient therapeutic state groups: subtherapeutic (data set 1: 79.35%; data set 2: 83.67%; data set 3: 83.33%), normal therapeutic (data set 1: 93.15%; data set 2: 87.76%; data set 3: 84.62%), and supratherapeutic (data set 1: 88.00%; data set 2: 86.54%; data set 3: 95.45%) therapeutic ranges, respectively.

**Conclusions:**

The most appropriate model for predicting the effects of heparin treatment was found by comparing multiple machine learning models and can be used to further guide optimal heparin dosing. Using multicenter intensive care unit data, our study demonstrates the feasibility of predicting the outcomes of heparin treatment using data-driven methods, and thus, how machine learning–based models can be used to optimize and personalize heparin dosing to improve patient safety. Manual analysis and validation suggested that the model outperformed standard practice heparin treatment dosing.

## Introduction

In hospitals, intensive care units are unique in that vast amounts of information are collected and displayed by computerized systems, and that the diagnostic and treatment accuracy can profoundly affect quality of care and patient outcomes [1]. Data-driven clinical decision support systems have the potential to help clinicians optimize treatment and medication in an intensive care unit to maximize the medical effect for each individual patient [2].

Heparin is one of the most commonly used medications in intensive care units, and intravenous unfractionated heparin is a fundamental method of anticoagulant therapy. In most clinical practice guidelines, heparin dosing is based only on the patient’s weight; the use of a weight-based heparin dosing nomogram is the standard practice for the treatment of thrombosis [3,4]. For patients who are obese who may not receive the appropriate heparin dose if it is determined based solely on body weight, some suggestions such as reducing the initial infusion rate [5-7] or using an adjusted body weight [8] have been reported. In clinical practice, activated partial thromboplastin time typically reflects blood coagulation level. A high activated partial thromboplastin time means that blood is clotting slowly, whereas a low activated partial thromboplastin time means that blood is clotting quickly. Typically, blood samples are drawn every 4 to 6 hours to monitor activated partial thromboplastin time, and the anticoagulation therapy outcome is measured by whether the activated partial thromboplastin time reaches the therapeutic window in a timely manner; however, the weight-based method easily leads to improper doses which demonstrate subtherapeutic or supratherapeutic activated partial thromboplastin time. In addition, the risk factors that result from inappropriate doses of unfractionated heparin are unclear. Only high initial rates of infusion, advanced age, and being female have been reported to be associated with supratherapeutic activated partial thromboplastin time [9,10]. Heparin administration guidelines regarding initial loading dose, maintenance dose and rate, and the activated partial thromboplastin time measurement intervals vary widely among institutions. Additionally, clinicians choose different heparin administration routes such as intravenous push or intravenous drip due based on the immediate circumstances and requirements of the patient.

Recently, machine learning techniques have dramatically improved the ability of computers to provide clinical decision support, resulting in the possibility of computer generated, algorithm-based heparin dosing recommendations. Multivariate logistic regression [11] and multinomial logistic regression [12] have been used to estimate heparin dosing with an accuracy of approximately 60%. Algorithms have also been used in studies [13,14] for other anticoagulants such as warfarin dose adjustments, but it was found that high intrapatient variability weakened the prediction accuracy.

For these reasons, a reliable method that can help doctors quickly predict and optimize heparin doses is urgently needed. It is necessary that modeling and prediction of the therapeutic window of activated partial thromboplastin time take into account multiple factors during patient treatment in order to provide appropriate decision support suggestions which can help guide clinicians in determining and preparing subsequent heparin doses or adjusting dose rate.

## Methods

### Data Set

Data were extracted from the Multiparameter Intelligent Monitoring In Intensive Care III database (MIMIC-III) [15] and e–Intensive Care Unit Collaborative Research database (eICU) [16] with the goal of comparing multiple predictive models and evaluating the results in different groups of patients. A cross-database evaluation was conducted. The MIMIC-III database and eICU database are free and open data sets containing medical data. The MIMIC-III database contains data from the intensive care unit at the Beth Israel Deaconess Medical Center and is published by the Laboratory for Computational Physiology at Massachusetts Institute of Technology. The eICU database, published by the Philips e–Intensive Care Unit Research Institute, is populated with data from a combination of many critical care units throughout the continental United States. Data were extracted from the databases for 14,806 adult patients who received heparin therapy during their stay in the intensive care unit. Only patient data with activated partial thromboplastin time measurements taken 4 to 6 hours after their initial heparin dose administration were used which reduced the cohort size to 3835. We chose 4 to 6 hours based on past experience and previous research [11]; it is the period within which the first activated partial thromboplastin time measurement typically occurred for the greatest proportion of patients. In clinical practice, there are different administration routes to deliver medication. Both intravenous push and intravenous drip are commonly used to deliver heparin, and in practice, are chosen based on patient condition and doctor preference; therefore, patient data were further classified by administration route—intravenous push (data set 1) and intravenous drip (data sets 2 and 3).

### Feature Selection

The outcome of interest was activated partial thromboplastin time 4 to 6 hours after initial heparin infusion. Since the data were from the Beth Israel Deaconess Medical Center, we applied the definition of therapeutic time used at Beth Israel Deaconess Medical Center for the definition of therapeutic time of activated partial thromboplastin time in this study to ensure consistency. Normal therapeutic was defined as activated partial thromboplastin times from 60 seconds to 100 seconds, supratherapeutic was defined as activated partial thromboplastin times greater than 100 seconds, and subtherapeutic was defined as activated partial thromboplastin times less than 60 seconds [11]. Clinical features of interest were selected to optimize the prediction of the therapeutic activated partial thromboplastin time—age, ethnicity, gender, initial heparin dose, interval between initial heparin injection and first measurement of activated partial thromboplastin time, creatinine concentration, type of admission, and the aspartate aminotransferase to alanine aminotransferase ratio (AST/ALT ratio). These features contribute as a whole to patient outcomes, for example, creatinine in the blood is almost entirely filtered into the urine via glomerular filtration, and its concentration is stable under normal circumstances; therefore, creatinine concentration in the blood can be used as an indicator of renal function because it reflects the filtration function of glomeruli. Aspartate aminotransferase and alanine aminotransferase concentration levels in the blood are sensitive to hepatocellular damage, and their ratio is an important indicator of liver function. These features have been reported and discussed in another study [11], and many of the features exhibited statistically significant relationships with the first measurement of activated partial thromboplastin time after initial heparin dose.

### Data Preprocessing

Patient data were preprocessed, and the features of interest were coded and normalized as variables. Missing values for some features were filled using the *k*–nearest neighbors algorithm which uses Euclidean distance to fill in missing values based on the values of its nearest neighbors in *k* dimensions.

Extreme values in data affect both the training and prediction processes. Normalization is needed when preprocessing continuous features; however, extreme values, though they may be few, negatively affect the output of normalization. Continuous features (age, heparin dose, creatinine value, and AST/ALT ratio) were manually verified to have *z* scores within the range of –3 to +3. According to the statistical definition of outliers [17], the normal range should be from *z*=−3 to *z*=+3; therefore, *z* scores outside of this range should be removed prior to normalization. Age data were found to be within the normal range; however, outliers were removed from initial heparin dose, creatinine concentration, and AST/ALT ratio data.

### Model Training and Performance Tuning

The activated partial thromboplastin time value measured 4 to 6 hours after the initial heparin dose was classified using ternary classification into sub, normal, and supratherapeutic. The support vector machine, random forest, adaptive boosting, extreme gradient boosting, and shallow neural network algorithms were implemented and tested in this study.

A support vector machine is based on maximization of the margin (ie, the minimum distance from the separating hyperplane to the nearest data point) between 2 classes of data. A Gaussian kernel guarantees that classification is nonlinear. Adaptive boosting, extreme gradient boosting, and random forest methods are based upon the use of boosting as the method of learning. Boosting methods select features that are known to improve model predictive power, and thus simultaneously, to reduce dimensionality. Where typically sample features are the outputs of a weak classifier that has been applied to each sample, adaptive boosting trains different weak classifiers by changing the weight of the samples, and the weak class is combined into a weighted sum that represents the final output of the boosted classifier. Extreme gradient boosting is based on gradient boosting, a process in which the algorithm learns an ensemble of boosted trees and makes a careful tradeoff between the classification error and model complexity. Extreme gradient boosting has recently become dominant in the field of applied machine learning (for example, in Kaggle competitions for structured or tabular data) [18]. The random forest method grows multiple decision trees, each of which provides a classification. The forest chooses the final output by the classification that has the majority. Artificial neural networks are built of multiple layers of neurons; each neuron receives a number of input variables and passes on the results to neurons in the next layer. An artificial neural network can learn complex functions relating input to output variables and is able to deal with complex relationships between variables and functions. Our shallow neural network was built using TensorFlow (version 1.13.1).

Samples from subtherapeutic, normal therapeutic, and supratherapeutic data groups were included at a 1:1:1 ratio for training and validation of the ternary classification model. Each data set was divided into 80% training and cross-validation and 20% testing.

The best parameters for the support vector machine, random forest, adaptive boosting, and extreme gradient boosting algorithms were searched (GridSearch; scikitlearn package) and used to train the models. In the shallow neural network model, 2 hidden layers were used, and the number of neurons was set at 36/24 to reduce model complexity. To avoid overfitting, early stopping and regularization were needed. Dropout was also used since it is an effective method to avoiding overfitting and to improve robustness. The rectified linear unit activation function was chosen to increase nonlinearity [16,17,19]. The Adam optimizer was used in model training with an initial learning rate of 0.0015. We trained the model for 1500 epochs with the dropout rate set at 0.75. To validate the predictive performance of our models, 5-fold cross-validation was used on each.

### Model Evaluation

The following measures, *precision* = *true positive*/(*true positive* × *false positive*), *recall* = *true positive*/(*true positive* + *false negative*), *F1 score* = 2 × (*precision* × *recall*)/(*precision* + *recall*), and *accuracy* = (*true positive* + *true negative*)/( *true positive* + *true negative* + *false positive* + *false negative*), were used to evaluate the capability of our 3-class classification model [20]. For samples at a ratio of 1:1:1, the microaveraged precision, recall, and F1 score are all equal to the accuracy; therefore, we only compared the average accuracy and macroaveraged precision, recall, and F1 score to gauge the classification performances of these models.

## Results

### Activated Partial Thromboplastin Time Distribution in the Study Population

**Figure 1:**
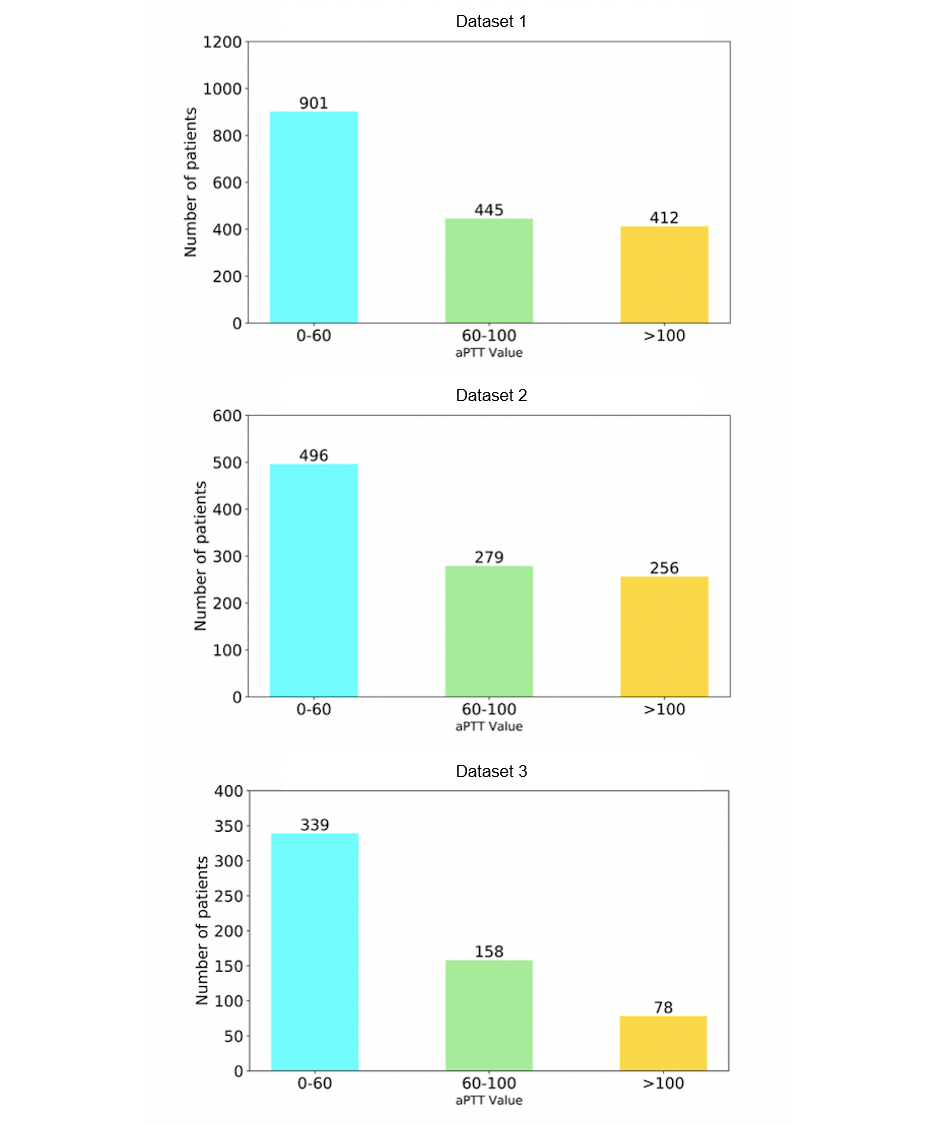
Patient distribution of aPPT value after initial heparin dosing.

After removing outliers, we extracted data on intravenous push patients (data set 1, n=1758) and intravenous drip patients (data set 2, n=1031) who met our inclusion criteria from the MIMIC-III database and data on intravenous drip patients (data set 3, n=575) from the eICU database, respectively. In data set 1, 25.3% (445/1758) of patients had measured values of activated partial thromboplastin time within the normal therapeutic range, 51.3% (901/1758) had measured values of activated partial thromboplastin time within the subtherapeutic range, and 23.4% (412/1758) had measured values of activated partial thromboplastin time within the supratherapeutic range. In data set 2, 27.0% (279/1031), 48.1% (496/1031), and 24.9% (256/1031) of patients had measured values of activated partial thromboplastin time within the normal, subtherapeutic, and supratherapeutic ranges, respectively, as shown in Figure 1. In data set 3, 27.6% (158/575), 59.0% (339/575), and 13.6% (78/575) of patients had measured values of activated partial thromboplastin time within the normal, subtherapeutic, and supratherapeutic ranges, respectively.

### Summary Statistics of Selected Features

A descriptive summary of patient data in data set 1, 2, and 3 according to the therapeutic range of the first measurement of activated partial thromboplastin time after the initial heparin injection is shown in Table 1.

**Table 1:** Summary statistics of selected features.

Patient groups and features			Therapeutic range		
			Sub	Normal	Supra
**Data set 1: MIMIC-III^a^ intravenous push (N=1756), n**			901	445	412
	Age (years), mean (SD)		65.4 (14.6)	68.1 (15.1)	69.3 (14.2)
	Initial heparin dose (units/hour), mean (SD)		907.0 (818.8)	1224.2 (1097.5)	1303.5 (908.4)
	aPTT^b^ (hours), mean (SD)		4.9 (0.6)	4.9 (0.6)	4.9 (0.6)
	**Ethnicity, n (%)**				
		White	639 (70.9)	311 (69.9)	291 (70.6)
		Asian	11 (1.2)	5 (1.1)	7 (1.7)
		Black	40 (4.4)	27 (6.1)	46 (11.2)
		Hispanic/Latino	13 (1.4)	11 (2.5)	14 (3.4)
		Others	198 (22.0)	91 (20.4)	54 (13.1)
	**Gender, n (%)**				
		Male	550 (61.0)	256 (57.5)	217 (52.7)
		Female	351 (39.0)	189 (42.5)	195 (48.3)
	**Admission type, n (%)**				
		Elective	111 (12.3)	26 (5.8)	15 (3.6)
		Emergency	768 (79.8)	398(89.4)	388 (94.2)
		Urgent	32 (3.6)	21 (4.7)	9 (2.2)
**Data set 2: MIMIC-III intravenous drip (N=1031), n**			496	279	256
	Age (years), mean (SD)		64.9 (15.4)	68.6 (15.2)	70.1 (14.8)
	Initial heparin dose (units/hour), mean (SD)		969.4 (398.3)	1148.7 (395.8)	1229.8 (495.3)
	aPTT (hours), mean (SD)		5.0 (0.6)	4.9 (0.5)	5.0 (0.6)
	**Ethnicity, n (%)**				
		White	353 (71.2)	208 (74.6)	179 (70.0)
		Asian	9 (1.8)	9 (3.2)	10 (3.9)
		Black	46 (9.3)	29 (10.4)	42 (16.4)
		Hispanic/Latino	12 (2.4)	7 (2.5)	9 (3.5)
		Others	76 (15.3)	26 (9.3)	15 (6.2)
	**Gender, n (%)**				
		Male	312 (62.9)	163 (58.4)	132 (51.6)
		Female	184 (37.1)	116 (41.6)	124 (48.4)
	**Admission type, n (%)**				
		Elective	59 (11.9)	25 (9.0)	8 (3.1)
		Emergency	436 (87.9)	250 (89.6)	245 (95.7)
		Urgent	1 (0.2)	4 (1.4)	3 (1.2)
**Data set 3: eICU^d^ intravenous drip (N=575), n**			339	158	78
	Age (years), mean (SD)		64.8 (13.9)	69.0 (14.4)	73.1 (12.3)
	Initial heparin dose (units/hour), mean (SD)		1005.7 (892.6)	973.5 (519.3)	950.4 (539.4)
	aPTT (hours), mean (SD)		5.2 (0.6)	5.2 (0.6)	5.2 (0.6)
	**Ethnicity, n (%)**				
		White	244 (72.0)	106 (67.1)	46 (59.0)
		Asian	4 (1.2)	2 (1.3)	2 (2.6)
		Black	30 (8.8)	20 (12.7)	9 (11.5)
		Hispanic/Latino	22 (6.5)	19 (12.0)	12 (15.4)
		Others	39 (11.5)	11 (7.0)	9 (11.5)
	**Gender, n (%)**				
		Male	217 (64.3)	99 (62.7)	37 (47.4)
		Female	122 (35.7)	59 (37.3)	41 (52.6)
	Creatinine (mg/dL), mean (SD)		1.7 (1.7)	2.0 (2.1)	2.0 (1.5)
	AST/ALT^c^, mean (SD)		1.5 (1.2)	1.7 (1.3)	1.5(1.1)

^a^Multiparameter Intelligent Monitoring In Intensive Care III database.

^b^First measurement of activated partial thromboplastin time.

^c^AST/ALT: aspartate aminotransferase ratio/alanine aminotransferase.

^d^eICU: e–Intensive Care Unit database.

### Data Preprocessing Results

Outliers were removed for 3 features: heparin dose, creatinine value, and AST/ALT ratio. The statistical outliers are shown in Multimedia Appendix 1. Not all patients had a complete set of clinical data, for example, 154 patients were missing AST/ALT ratios, accounting for 8.76% of intravenous push patients (Multimedia Appendix 2). An algorithm (*k* nearest neighbors) was used to fill in the missing values. Since filled values accounting for up to 40% have been reported to be appropriate [21], we considered the effect of filled features on the activated partial thromboplastin time as reasonable.

### Model Performance Results

To eliminate category imbalances, we randomly selected 400 samples for each therapeutic state in data set 1, 250 samples for each therapeutic state in data set 2, and 120 samples for each therapeutic state in data set 3. For subtherapeutic and normal therapeutic classes, general downsampling was used to reduce the number of samples, while for the supratherapeutic class we used upsampling to increase the number of samples to 120; therefore, experiments used 1200 samples from data set 1, 750 samples from data set 2, and 360 samples from data set 3. Model performance results are shown in Table 2.

The F1 score provides a comprehensive evaluation of the model. As listed in Multimedia Appendix 3, extreme gradient boosting achieved the second best F1 scores (77.58%, 73.94%, and 78.85% for data set 1, 2, and 3, respectively), second only to those of the shallow neural network (87.26%, 85.98% and 87.55% for data set 1, 2, and 3, respectively). The adaptive boosting model also performed very well in all 3 data sets (72.80%, 81.67%, and 77.65% for data set 1, 2, and 3, respectively), with scores close to those of extreme gradient boosting (77.58%, 73.94%, and 78.85% for data set 1, 2, and 3, respectively). The random forest performed slightly worse (68.20%, 73.15%, and 65.59% for data set 1, 2, and 3, respectively) than the other 4 models. The confusion matrices of all 5 models are shown in Multimedia Appendix 4. In further experiments, the random forest still performed better than other models that were not discussed herein, such as the Naïve Bayes, logistic regression, *k* nearest neighbors, and decision tree, as shown in Multimedia Appendix 3.

**Table 2:** Macroaveraged scores for the machine learning algorithms.

Models		Precision, %	Recall, %	F1 score, %	Accuracy, %
**Data set 1: MIMIC-III^a^ (intravenous push patients)**					
	Random forest	68.96	68.75	68.70	68.75
	Adaptive boosting	74.37	72.92	72.80	72.92
	Support vector machine	85.19	73.33	73.79	73.33
	Extreme gradient boosting	79.27	76.25	77.58	76.25
	Shallow neural network	88.05	86.67	87.26	86.67
**Data set 2: MIMIC-III (intravenous drip patients)**					
	Random forest	66.71	65.33	65.06	65.33
	Adaptive boosting	77.29	77.33	77.30	77.33
	Support vector machine	84.59	71.33	71.71	71.33
	Extreme gradient boosting	77.45	77.33	77.38	77.33
	Shallow neural network	85.99	86.00	85.98	86.00
**Data set 3: eICU^b^ (intravenous drip patients)**					
	Random forest	66.77	66.56	65.59	68.06
	Adaptive boosting	78.03	77.78	77.65	77.78
	Support vector machine	84.74	76.39	76.19	76.39
	Extreme gradient boosting	79.16	79.17	78.85	79.17
	Shallow neural network	87.80	87.50	87.55	87.50

^a^Multiparameter Intelligent Monitoring In Intensive Care III database.

^b^eICU: e–Intensive Care Unit database.

In the subtherapeutic class, adaptive boosting achieved the highest precision in data set 1 (84.48%) while the neural network model achieved highest in the other data sets (data set 2: 83.67%; data set 3: 83.33%). The support vector machine achieved the highest recall in all 3 data sets (data set 1: 100%; data set 2: 100%; data set 3: 95.83%). In the normal therapeutic class, the support vector machine with the Gaussian kernel achieved 100% precision in all 3 data sets. The shallow neural network achieved the highest recall (data set 1: 85.00%; data set 2: 86.00%; data set 3: 91.67%). In the supratherapeutic class, the support vector machine achieved the highest precision (data set 1: 100%; data set 2: 100%; data set 3: 95.24%); however, recall of the support vector machine was not very high (data set 1: 57.50%; data set 2: 58.00%; data set 3: 83.33%). The shallow neural network achieved the best recall in all 3 data sets (data set 1: 100%; data set 2: 100%; data set 3: 95.83%). Considering the comprehensive performance which is best evaluated by F1 score, the shallow neural network achieved the best F1 score in all 3 patient groups: subtherapeutic (data set 1: 79.35%; data set 2: 83.67%; data set 3: 83.33%), normal therapeutic (data set 1: 93.15%; data set 2: 87.76%; data set 3: 84.62%), and supratherapeutic (data set 1: 88.00%; data set 2: 86.54%; data set 3: 95.45%) therapeutic ranges. Additional results are listed in Table 3, Table 4, and Table 5.

**Table 3:** Model performance for subtherapeutic.

Models		Precision, %	Recall, %	F1 score, %
**Data set 1: MIMIC-III^a^ (intravenous push patients)**				
	Random forest	67.61	60.00	63.58
	Adaptive boosting	84.48	61.25	71.01
	Support vector machine	55.56	100	71.43
	Extreme gradient boosting	74.32	68.75	71.43
	Shallow neural network	79.35	91.25	84.89
**Data set 2: MIMIC-III (intravenous drip patients)**				
	Random forest	58.82	80.00	67.80
	Adaptive boosting	78.43	80.00	79.21
	Support vector machine	53.76	100	69.93
	Extreme gradient boosting	74.32	68.75	71.43
	Shallow neural network	83.67	82.00	82.83
**Data set 3: eICU^b^ (intravenous drip patients)**				
	Random forest	66.67	58.33	62.22
	Adaptive boosting	77.27	70.83	73.91
	Support vector machine	58.97	95.83	73.02
	Extreme gradient boosting	76.00	79.17	77.55
	Shallow neural network	83.33	83.33	83.33

^a^Multiparameter Intelligent Monitoring In Intensive Care III database.

^b^eICU: e–Intensive Care Unit database.

**Table 4:** Model performance for normal therapeutic.

Models		Precision, %	Recall, %	F1 score, %
**Data set 1: MIMIC-III^a^ (intravenous push patients)**				
	Random forest	63.64	84.00	72.41
	Adaptive boosting	71.26	77.50	74.25
	Support vector machine	100	62.50	76.92
	Extreme gradient boosting	78.72	74.00	76.29
	Shallow neural network	93.15	85.00	88.89
**Data set 2: MIMIC-III (intravenous drip patients)**				
	Random forest	73.81	62.00	67.39
	Adaptive boosting	78.43	80.00	79.21
	Support vector machine	100	56.00	71.79
	Extreme gradient boosting	72.55	74.00	73.27
	Shallow neural network	87.76	86.00	86.87
**Data set 3: eICU^b^ (intravenous drip patients)**				
	Random forest	70.00	65.00	61.90
	Adaptive boosting	81.82	53.85	78.26
	Support vector machine	100	50.00	66.67
	Extreme gradient boosting	80.00	66.67	72.73
	Shallow neural network	84.62	91.67	87.50

^a^Multiparameter Intelligent Monitoring In Intensive Care III database.

^b^eICU: e–Intensive Care Unit database.

**Table 5:** Model performance for supratherapeutic.

Models		Precision, %	Recall, %	F1 score, %
**Data set 1: MIMIC-III^a^ (intravenous push patients)**				
	Random forest	75.95	75.00	75.47
	Adaptive boosting	67.37	80.00	73.14
	Support vector machine	100	57.50	73.02
	Extreme gradient boosting	80.77	78.75	79.75
	Shallow neural network	88.00	82.50	85.16
**Data set 2: MIMIC-III (intravenous drip patients)**				
	Random forest	67.50	54.00	60.00
	Adaptive boosting	75.00	72.00	73.47
	Support vector machine	100	58.00	73.42
	Extreme gradient boosting	76.47	78.00	77.23
	Shallow neural network	86.54	90.00	88.24
**Data set 3: eICU^b^ (intravenous drip patients)**				
	Random forest	63.64	87.50	73.68
	Adaptive boosting	75.00	87.50	80.77
	Support vector machine	95.24	83.33	88.89
	Extreme gradient boosting	81.48	91.67	86.27
	Shallow neural network	95.45	87.50	91.30

^a^Multiparameter Intelligent Monitoring In Intensive Care III database.

^b^eICU: e–Intensive Care Unit database.

## Discussion

### Principal Results

In our experiments, the neural network achieved the highest scores for all evaluation metrics. The neural network model uses multiple layers to progressively extract higher level features from the raw data which might be the reason that the neural network is able to learn some unknown features that help to provide a better classification of normal therapeutic activated partial thromboplastin time. Since different features may be correlated (such as the creatinine value and aspartate aminotransferase), linear classification models are not appropriate. Random forest, adaptive boosting, and extreme gradient boosting are ensemble learning methods. By integrating weak classifiers, classification performance was greatly improved. The support vector machine with Gaussian kernel is a widely used and powerful classifier. Gaussian kernels ensure that the classifier is nonlinear, which suited the characteristics of our data, and the method was able to demonstrate high performance; however, the neural network model was able to take into account complex relationships between the variables with complex functions. Among the methods tested, the shallow neural network performed the best. The shallow neural network achieved performance approximately 10% higher than that of the other algorithms for each metric (precision, recall, F1 score, and accuracy) in intravenous push cases (data set 1) and achieved performance approximately 9% higher than that of the other algorithm metrics in intravenous drip cases (data set 2 and data set 3). Extreme gradient boosting, adaptive boosting, and the support vector machine were the models that subperformed to the shallow neural network although their scores were, nevertheless, all above 70%. The random forest model demonstrated the worst performance.

As a result of its relative high accuracy, this shallow neural network model should be able to recommend doses better than the heparin dosage guidelines which only take patient weight into account.

In clinical practice, intravenous push and intravenous drip are both common delivery routes for heparin. Intravenous push heparin is always used to rescue critical patients who require timely intervention to decrease coagulation, while intravenous drip heparin is used a long-term medication to prevent thrombosis or embolic disease. These 2 administration routes have different clinical significance; therefore, we separated the patient groups from the 2 databases into 3 data sets to verify whether they would have different model predictions. The results suggested that model prediction performance was comparable among the 3 data sets, which gave us insight into the stability and suggests the model is stable regardless of administration routes or data source.

### Strengths

Since the range of normal therapeutic activated partial thromboplastin time varies in different institutions, our shallow neural network model can be adapted to different heparin administration guidelines by adjusting the parameters. Furthermore, the model can also be applied to other drug dosage optimization problems after retraining. When treating a patient, a dose of heparin can be recommended that maximizes the normal therapeutic probability. The future application of the model prediction has the potential to enhance patient safety, minimize the risk of bleeding or a thromboembolic event, reduce medical costs, and improve the efficiency of clinicians.

### Limitations

One challenge of our study was to identify the features that affect heparin doses. First, balancing both discrete features and continuous features and their relative importance would have enhanced model training performance and feature utilization but was not performed in this study. Second, different features may have been correlated, since they all contribute to the comprehensive conditions of patients; therefore, determining the intrinsic relationships would have further improved model performance. Model optimization and verification using different intensive care unit databases will be performed in future research. Drug interactions with heparin and the accumulated effects are usually not taken into account since the half-time of heparin is too short to affect the 4 to 6–hour interval that was monitored. A more precise neural network structure was not used; the next step would be to explore the intrinsic relationships between features and further validate the model results using additional clinical data sets. Since this study was conducted in a nonclinical setting, it will be further refined as it is used in practice.

### Comparison With Prior Work

It is difficult to obtain personalized rather than broad normative data to determine drug dosage in intensive care units. Heparin dose is commonly determined based solely upon body weight, which is measured or estimated when patients arrive at the intensive care unit. Here, we distinguished 2 drug delivery routes to provide more detailed advice and choices for clinicians. The overall prediction accuracies for the 3 data sets were 88.00%, 86.00%, and 87.50%. Both delivery routes in the MIMIC-III retrospective data showed proportions of patients with activated partial thromboplastin times that were 3-fold higher than those with normal therapeutic activated partial thromboplastin times (25.3% for intravenous push patients and 27.0% for intravenous drip patients), and higher than those reported in previous studies [11,12] for the multivariate logistic regression (volume under the surface=0.48) and multinomial logistic regression (accuracy=60%). Statistical results were consistent with those from previous reports. Advanced age and gender (female) were reported to be associated with supratherapeutic activated partial thromboplastin time [9,10], as well as a high initial heparin dose, a high AST/ALT ratio, and emergency admission-type.

### Conclusions

The study aimed to provide support to predict heparin treatment outcomes and recommend optimal heparin dosing to clinicians. Data-driven machine learning methods were used to predict the probabilities of subtherapeutic, normal therapeutic, and supratherapeutic activated partial thromboplastin time. After comparing different models, we recommend the adoption of a support system comprising a shallow neural network with parameter adjustability. The results of this study provide new insights into personalized medication optimization and demonstrate the feasibility of applying the model in different medical institutions.

## Appendix

Multimedia Appendix 1 Outliers preprocessing.

Multimedia Appendix 2 Missing data imputation.

Multimedia Appendix 3 Macroaveraged scores of different algorithms on 3 different datasets.

Multimedia Appendix 4 Confusion matrix.

## Abbreviations

ALT: alanine aminotransferase
AST: aspartate aminotransferase
eICU: e–Intensive Care Unit
MIMIC-III: Multiparameter Intelligent Monitoring in Intensive Care III (or Medical Information Mart for Intensive Care III)
