# Supplementary material for: Toward Optimal Heparin Dosing by Comparing Multiple Machine Learning Methods: Retrospective Study
Source: JMIR Med Inform. 2020 Jun 22;8(6):e17648. doi: 10.2196/17648 (PMC7338927; doi:10.2196/17648)
Supplement: Multimedia Appendix 1 [file medinform_v8i6e17648_app1.docx]

Appendix 1: Outliers preprocessing

|  | Features | Mean | Std | min | max | Normal range | Outliers |
| --- | --- | --- | --- | --- | --- | --- | --- |
| **Dataset 1** | **Heparin Dose** | 1198.7 | 2756.1 | 3 | 85000 | (0, 9746.1) | 13 |
|  | **Creatinine Value** | 1.6 | 3.1 | 0.1 | 116.0 | (0, 10.8) | 5 |
|  | **AST/ALT** | 1.3 | 1.2 | 0 | 21.1 | (0, 4.9) | 22 |
| **Dataset 2** | **Heparin rate** | 1106.3 | 470.0 | 10.0 | 6437.7 | (0, 2516.4) | 11 |
|  | **creatinine value** | 1.6 | 31.6 | 0.2 | 22.2 | (0, 5.4) | 22 |
|  | **AST/ALT** | 1.3 | 4.8 | 0.4 | 157.5 | (0, 15.7) | 1 |
| **Dataset 3** | **Heparin rate** | 989.3 | 763.0 | 6.5 | 12884 | (0,3278.3) | 2 |
|  | **Creatinine Value** | 1.8 | 1.8 | 0.3 | 18.4 | (0, 7.2) | 10 |
|  | **AST/ALT** | 1.6 | 1.2 | 0.2 | 9.5 | (0, 5.3) | 12 |

Mean( ): mean value;

Std( ): standard deviation;

Min: minimal value;

Max: maximum value;

Normal range:

Outliers: values outside normal range
