# Supplementary material for: Toward Optimal Heparin Dosing by Comparing Multiple Machine Learning Methods: Retrospective Study
Source: JMIR Med Inform. 2020 Jun 22;8(6):e17648. doi: 10.2196/17648 (PMC7338927; doi:10.2196/17648)
Supplement: Multimedia Appendix 2 [file medinform_v8i6e17648_app2.docx]

Appendix 2. Missing data imputation

| Patient groups\Features | AST/ALT | creatinine |
| --- | --- | --- |
| **Dataset 1** | 154 | 3 |
| **Dataset 2** | 1 | 2 |
| **Dataset 3** | 11 | 1 |
