# Supplementary material for: Toward Optimal Heparin Dosing by Comparing Multiple Machine Learning Methods: Retrospective Study
Source: JMIR Med Inform. 2020 Jun 22;8(6):e17648. doi: 10.2196/17648 (PMC7338927; doi:10.2196/17648)
Supplement: Multimedia Appendix 3 [file medinform_v8i6e17648_app3.docx]

Appendix 3: Marco-average scores of different algorithms on 3 different datasets

| **Patient Groups** | **Models** | **precision** | **recall** | **F1-score** | **accuracy** |
| --- | --- | --- | --- | --- | --- |
| **Dataset 1** | Naive Bayes | 42.86% | 43.75% | 41.60% | 43.75% |
|  | Logistic regression | 47.98% | 49.17% | 48.05% | 49.17% |
|  | K-nearest neighbor | 59.11% | 59.33% | 59.11% | 59.33% |
|  | Decision tree | 61.39% | 61.25% | 61.29% | 61.25% |
|  | Random forest | 68.96% | 68.75% | 68.70% | 68.75% |
|  | Adaptive boosting | 74.37% | 72.92% | 72.80% | 72.92% |
|  | Support vector machine | 85.19% | 73.33% | 73.79% | 73.33% |
|  | Extremal gradient boosting | 79.27% | 76.25% | 77.58% | 76.25% |
|  | Shallow neural network | 88.05% | 86.67% | 87.26% | 88.00% |
| **Dataset 2** | Naive Bayes | 51.96% | 50.00% | 47.69% | 50.00% |
|  | Logistic regression | 55.00% | 54.00% | 52.18% | 54.00% |
|  | K-nearest neighbor | 59.92% | 59.58% | 59.62% | 59.58% |
|  | Decision tree | 63.53% | 62.67% | 62.65% | 62.67% |
|  | Random forest | 66.71% | 65.33% | 65.06% | 65.33% |
|  | Adaptive boosting | 77.29% | 77.33% | 77.30% | 77.33% |
|  | Support vector machine | 84.59% | 71.33% | 71.71% | 71.33% |
|  | Extremal gradient boosting | 77.45% | 77.33% | 77.38% | 77.33% |
|  | Shallow neural network | 85.99% | 86.00% | 85.98% | 86.00% |
| **Dataset 3** | Naive Bayes | 57.34% | 51.39% | 46.80% | 51.39% |
|  | Logistic regression | 57.77% | 55.56% | 55.26% | 55.56% |
|  | K-nearest neighbor | 54.17% | 54.17% | 54.17% | 54.17% |
|  | Decision tree | 65.34% | 63.89% | 64.01% | 63.89% |
|  | Random forest | 66.77% | 66.56% | 65.59% | 68.06% |
|  | Adaptive boosting | 78.03% | 77.78% | 77.65% | 77.78% |
|  | Support vector machine | 84.74% | 76.39% | 76.19% | 76.39% |
|  | Extremal gradient boosting | 79.16% | 79.17% | 78.85% | 79.17% |
|  | Shallow neural network | 87.80% | 87.50% | 87.55% | 87.50% |
