# Supplementary material for: Toward Optimal Heparin Dosing by Comparing Multiple Machine Learning Methods: Retrospective Study
Source: JMIR Med Inform. 2020 Jun 22;8(6):e17648. doi: 10.2196/17648 (PMC7338927; doi:10.2196/17648)
Supplement: Multimedia Appendix 4 [file medinform_v8i6e17648_app4.docx]

Appendix 4: Confusion matrix

|  | Dataset 1 | | | Dataset 2 | | | Dataset 3 | | |
| --- | --- | --- | --- | --- | --- | --- | --- | --- | --- |
| Random forest | 48 | 22 | 10 | 40 | 4 | 6 | 14 | 4 | 6 |
|  | 14 | 57 | 9 | 12 | 31 | 7 | 6 | 14 | 4 |
|  | 9 | 11 | 60 | 16 | 7 | 27 | 1 | 2 | 21 |
| Adaptive  boosting | 49 | 11 | 20 | 40 | 3 | 7 | 17 | 2 | 5 |
|  | 7 | 62 | 11 | 5 | 40 | 5 | 4 | 18 | 2 |
|  | 2 | 14 | 64 | 6 | 8 | 36 | 1 | 2 | 21 |
| Support vector machine | 80 | 0 | 0 | 50 | 0 | 0 | 23 | 0 | 1 |
|  | 30 | 50 | 0 | 22 | 28 | 0 | 12 | 12 | 0 |
|  | 34 | 0 | 46 | 21 | 0 | 29 | 4 | 0 | 20 |
| Extremal gradient boosting | 55 | 12 | 13 | 40 | 6 | 4 | 19 | 3 | 2 |
|  | 13 | 65 | 2 | 5 | 37 | 8 | 5 | 16 | 3 |
|  | 6 | 11 | 63 | 3 | 8 | 39 | 1 | 1 | 22 |
| Shallow neural network | 73 | 2 | 5 | 41 | 5 | 4 | 20 | 3 | 1 |
|  | 8 | 68 | 4 | 4 | 43 | 3 | 2 | 22 | 0 |
|  | 11 | 3 | 66 | 4 | 1 | 45 | 2 | 1 | 21 |
